# Investigation of a methylated *BCAT1* and *IKZF1* circulating tumour DNA blood test for monitoring treatment response in patients with colorectal cancer

**DOI:** 10.1007/s00432-026-06512-x

**Published:** 2026-06-02

**Authors:** Rafha Rafeeu, Erin L. Symonds, Michael Z. Michael, Kathryn Cornthwaite, Susanne K. Pedersen, Renee J. Smith, Graeme P. Young, Jean M. Winter

**Affiliations:** 1 Flinders Health and Medical Research Institute, College of Medicine and Public Health, Flinders University of South Australia, Bedford Park, SA 5042 Australia; 2 Gastroenterology Department, Flinders Medical Centre, Bedford Park, Australia SA 5042

**Keywords:** Liquid biopsy, Personalised treatment monitoring, Non-invasive biomarkers

## Abstract

**Purpose:**

Colorectal cancer (CRC) treatment failure results in high mortality. The current CRC treatment response monitoring blood biomarker carcinoembryonic antigen (CEA) lacks specificity. Methylated *BCAT1/IKZF1* circulating tumour DNA (ctDNA) indicates CRC, but its utility during therapy is uncertain. This study examined whether during-treatment measurement of *BCAT1/IKZF1* ctDNA reflects CRC treatment efficacy.

**Methods:**

Plasma was collected from CRC patients before and during chemotherapy, targeted therapy and/or radiotherapy. Cell-free DNA was extracted, bisulphite-converted and assayed via multiplex qPCR for *BCAT1/IKZF1* methylation (%pg/*ACTB*). Patients with detectable ctDNA pre-treatment were included. CEA was measured via chemiluminescent immunoassay. Radiological treatment response assessments were performed ± 3 months of blood collection or < 8 months post-treatment. Biomarker levels were compared using Mann–Whitney U and Kruskal–Wallis tests.

**Results:**

56 samples from 29 patients (64.3% male, median age 55 years) were analysed. Patients with residual cancer had higher ctDNA levels (median 0.036%, IQR 0–1.17%) than cancer-free patients (0%, IQR 0–0.005%; *p* < 0.05). There were no significant differences for CEA. When normalised to pre-treatment measurements, treatment-responders demonstrated a median 100% ctDNA reduction from baseline (IQR − 100% to − 7.53%), which was greater than non-responders (0.13% reduction, IQR − 38.1% to + 0.03%; *p* < 0.001). Responders also demonstrated greater CEA reduction (median − 1.50 ng/mL, IQR − 6.28 to 0.18 ng/mL) compared to non-responders (median 2.3 ng/mL, IQR − 0.50 to 17.7 ng/mL; *p* = 0.001).

**Conclusions:**

Methylated *BCAT1*/*IKZF1* ctDNA reflects treatment response, offering a tool for personalised treatment.

**Supplementary Information:**

The online version contains supplementary material available at 10.1007/s00432-026-06512-x.

## Introduction

Colorectal cancer (CRC) is the third most commonly diagnosed cancer and second leading cause of cancer-related death worldwide (Bray et al. 2024). Standard of care treatment for CRC involves surgical resection alone for patients with stage I/low-risk stage II disease, with the addition of neoadjuvant/adjuvant chemotherapy, radiotherapy and/or targeted therapies for patients with high-risk stage II, stage III and resectable stage IV disease (Cancer Council Australia 2024). Despite improvements in treatment efficacy in recent years, there are still relatively high recurrence rates of 12% for stage II CRC and 25% for stage III CRC (Nors et al. 2024), and individuals with stage IV CRC have a 5-year relative survival rate of only 16% (National Cancer Insitute n.d.). Overall, the high recurrence rates and mortality reflect a deficit in CRC treatment. Accurately monitoring response to treatment is crucial, as optimising treatment based on response can potentially prevent or reduce cancer progression and recurrence.

Standardised guidelines on CRC treatment response monitoring are lacking for high-risk stage II and stage III CRC. Monitoring is generally conducted via radiological imaging modalities such as chest, abdomen and pelvis CT-scans, MRI and PET scans (McKeown et al. 2014), with these measurements recommended as often as every 12 weeks for stage IV disease (Yoshino et al. 2023). Despite being important tools for accurately detecting cancer, radiological imaging techniques result in radiation exposure and high costs, along with a risk for late detection of disease progression if not conducted frequently enough (Wille-Jørgensen et al. 2018; Kirsten Gormly et al. 2024). In comparison, non-invasive biomarker tests are relatively cheaper and less resource-intensive, presenting as a possible alternative or addition to imaging. Indeed, the blood biomarker carcinoembryonic antigen (CEA) is often used alongside routine imaging for monitoring of CRC treatment response, with a sensitivity of 50–80% and specificity of 80–98% for recurrent CRC (Kirsten Gormly et al. 2024; Sørensen et al. 2016). However, CEA can be elevated in non-malignant conditions such as ulcerative colitis, chronic obstructive pulmonary disease and pancreatitis, as well as in smokers (Ha et al. 2019). Accurate biomarkers for CRC treatment response monitoring are therefore needed.

Circulating tumour DNA (ctDNA) blood tests that assay for cancer-related genetic and epigenetic alterations are promising alternatives for CRC treatment monitoring. For example, Tie et al. (2015) found in a cohort of patients with stage IV CRC, that reduction of mutation-based ctDNA during adjuvant chemotherapy (relative to pre-treatment levels) correlated with radiologic response in 74% of patients. Similarly, Urbini et al. (2023) found that an increase in mutation-based ctDNA during treatment of stage IV CRC preceded radiological progression in 87.5% of patients. However, the high genetic heterogeneity of CRC is a barrier in the widespread clinical application of mutation-based ctDNA assays (Molinari et al. 2018). Tissue utilised for genotyping may not accurately reflect the true tumour mutation profile due to intra-tumour heterogeneity, while clonal shift allows for the dominant tumour mutation profile to change over time and treatment (Molinari et al. 2018). Additionally, tumour biopsy may not always be feasible, due to the tumour location and risk of metastatic seeding (Bronkhorst and Holdenrieder 2023). For broader applicability, epigenetic alterations that are homogenous across all CRC types, such as DNA hypermethylation, may be more suitable.

Our previous work has shown that over 90% of CRC tumour tissue express DNA hypermethylation of either *BCAT1* or *IKZF1* (Symonds et al. 2018), with detection in plasma as ctDNA showing high sensitivity for CRC (62–66%) (Symonds et al. 2018; Pedersen et al. 2015). The detection of methylated *BCAT1/IKZF1* ctDNA after completion of curative-intent treatment is significantly associated with residual disease (Symonds et al. 2018). Additionally, the ctDNA assay can more accurately detect recurrence than CEA, with a sensitivity of 63–68% compared to 32–48% (Pedersen et al. 2023; Young et al. 2016; Symonds et al. 2020, 2018; Musher et al. 2020).

However, to be useful in timely patient management, it is important to assess whether measurements of methylated *BCAT1/IKZF1* ctDNA during treatment can accurately reflect tumour response to treatment, to ensure appropriate clinical intervention. Therefore, we report a prospective observational study that determined whether methylated *BCAT1/IKZF1* ctDNA sampled during treatment can accurately identify response to treatment in patients with CRC undergoing chemotherapy, targeted therapy and/or radiotherapy.

## Materials and methods

### Study population

Individuals > 18 years diagnosed with AJCC/UICC stage II, III or IV colorectal adenocarcinoma were approached by clinical research nurses at Flinders Medical Centre or Queen Elizabeth Hospital, South Australia, and informed consent was obtained. Blood was collected opportunistically at scheduled clinical appointments before and during treatment (chemotherapy, targeted and/or radiotherapy), and assayed for methylated *BCAT1/IKZF1* ctDNA. The patients selected for this study were those who were found to be positive for methylated *BCAT1/IKZF1* ctDNA before treatment and had at least one blood collection during treatment.

The study was approved by the Southern Adelaide Clinical Human Research Ethics Committee (reference number 134.045), registered in the Australia and New Zealand Clinical Trials Registry (registration number #12611000318987), and conducted in accordance with the standards set by the Declaration of Helsinki.

### Blood collection and processing

Venous blood was collected from patients into 9 mL K3EDTA Vacuette tubes (Greiner Bio-One, Frickenhausen, Germany), placed on ice and processed within 4 h of collection at the analytical laboratory. Blood was centrifuged at 1500 rcf for 12 min at 4 °C and the plasma fraction was retained, followed by repeat centrifugation with the same settings. 3.9–4.5 mL of plasma was stored at − 80 °C before transport for extraction of cell-free DNA (cfDNA). From the same blood sample, 400 μL of plasma was collected and transported for CEA analysis.

### DNA methylation analysis

cfDNA was extracted on the QIASymphony SP (Qiagen, Hilden, Germany), using the QIASymphony DSP DNA Midi kit (96) (Qiagen), and eluted in 60 μL nuclease free water. The cfDNA was bisulphite-converted on the QIACube HT (Qiagen), using the Epitect Fast Bisulphite Conversion kit (Qiagen) as previously described (Symonds et al. 2018).

Bisulphite converted cfDNA samples were assayed in triplicate by multiplex quantitative PCR targeting a fully methylated region in *BCAT1*, a partially methylated region in *IKZF1* and *ACTB* as the reference gene, as per previous research (Symonds et al. 2016). Quantitative levels of methylated *BCAT1*/*IKZF1* ctDNA were expressed as the sum of the mass of methylated *BCAT1* and/or *IKZF1* ctDNA as a percentage of total mass measured by *ACTB* (% pg/*ACTB*) and was averaged over the triplicates. As it is known that ctDNA levels vary with cancer stage (Symonds et al. 2016), quantitative ctDNA levels were also normalised to pre-treatment measurements (% pre-treatment concentration). Samples were deemed positive if methylated *BCAT1* and/or *IKZF1* was detected in at least one PCR replicate within 50 cycles.

### CEA analysis

CEA concentration in plasma was analysed on the Alinity i Immunoassay analyser (Abbott), with samples deemed CEA-positive if levels were higher than the reference normal 8 ng/mL. Where matched plasma aliquots were not available for CEA assessment due to insufficient volume of plasma available for analysis, serum CEA levels assessed for clinical purposes were determined from patient medical records that were within ± 1 month of ctDNA assessment.

### Treatment response classification

Diagnostic tumour staging, treatment response and cancer recurrence information for each patient were collected from surgical histopathology reports, radiology (CT, PET scans, MRI) reports, and colonoscopy reports. Diagnostic staging was determined at surgery or pre-treatment MRI. Clinicopathological data collected included the AJCC/UICC staging, TNM staging, primary cancer location and metastatic location. Primary cancer site locations were defined as proximal (caecum, ascending colon, hepatic flexure and transverse colon), distal (splenic flexure, descending colon and sigmoid colon), rectosigmoid, or rectal (Yang et al. 2016). Recurrence was defined as local (at the anastomosis), regional (in the lymph nodes) or distant (in a different area to the original tumour). Treatment response was classified as a complete response, partial response, stable or progression, based on clinicopathologic findings within ± 3 months of blood collection during treatment, or within 8 months after treatment cessation. Response to treatment was also further classified into 1) complete response (disease not present) vs. incomplete response (disease present), or 2) complete/partial response (responders) vs. stable/progressive disease (non-responders). ctDNA and CEA test results were blinded during treatment response assessment.

### Statistical analysis

Quantitative methylated ctDNA and CEA levels were expressed as median with interquartile range (IQR). Data was assessed for normality via the D’Agustino-Pearson test. ctDNA and CEA levels, and the change in levels between consecutive blood collections, were compared between treatment response groups via Mann–Whitney U tests, or Kruskal–Wallis tests followed by Dunn’s tests for multiple pair-wise comparisons. Univariate analysis of factors associated with disease presence, ctDNA positivity and CEA positivity were conducted via chi-squared tests or Fisher’s exact tests. Multivariate analysis was conducted via multivariable binomial logistic regression analysis (expressed as odds ratio (OR) with 95% confidence intervals (CI)). Statistical analyses were performed using GraphPad Prism v8.0.2 (GraphPad Software Inc, United States) and Stata 15 (StataCorp LLC, United States). Statistical significance was deemed at *p* < 0.05.

## Results

### Population characteristics

**Fig. 1:**
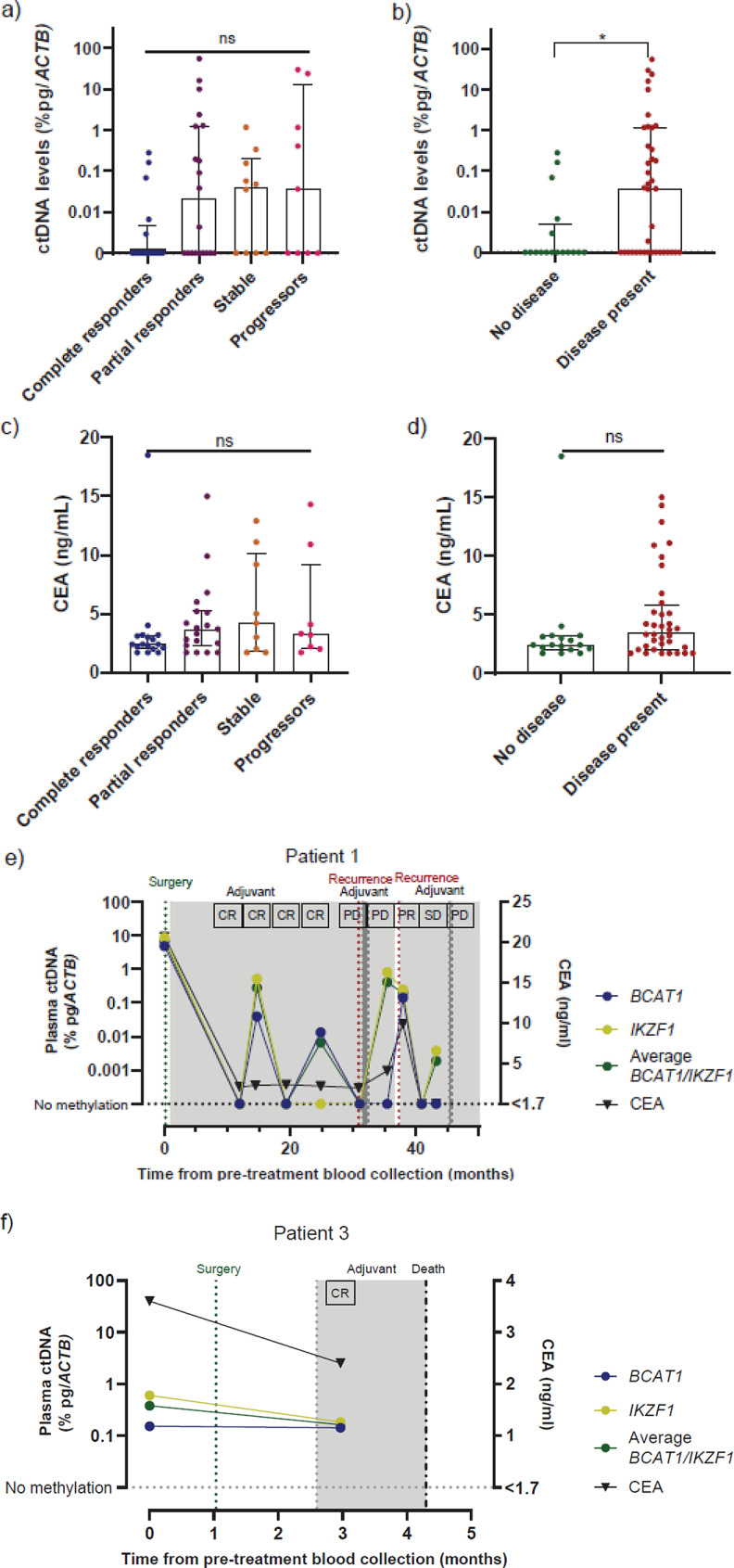
**a** ctDNA levels (%pg/*ACTB*) during treatment at the time of complete response, partial response, stable disease and disease progression. **b** ctDNA levels (%pg/*ACTB*) during treatment in patients with disease present vs. no disease. **c** CEA levels (ng/mL) during treatment in complete responders, partial responders, stable patients and progressors. **d** CEA levels (ng/mL) during treatment in patients with disease present vs. no disease. **e** Patient 1 methylated *BCAT1* ctDNA levels, methylated *IKZF1* ctDNA levels, average methylated *BCAT1/IKZF1* ctDNA levels and CEA levels across treatment. Methylated *BCAT1* = dark blue circle, methylated *IKZF1* = yellow circle, average methylated *BCAT1/IKZF1* = green circle, black triangle = CEA. Chemotherapy ± targeted therapy and targeted therapy only = light grey shading, radiotherapy = dark grey shading. Surgery = green dotted line, recurrence = red dotted line and death = black dotted line. Treatment response status is indicated above each ctDNA blood collection point (CR = complete response, PR = partial response, SD = stable disease and PD = progressive disease). **f** Patient 3 methylated *BCAT1* ctDNA levels, methylated *IKZF1* ctDNA levels, average methylated *BCAT1/IKZF1* ctDNA levels and CEA levels across treatment. *p < 0.05, Mann–Whitney U tests

There were 29 patients who tested ctDNA positive before treatment and from whom 56 blood samples were collected during treatment (Table 1). Patients were followed up for a median 22 months (IQR 13.6–30.3 months) from diagnosis to last reference clinicopathologic report. The median time between blood collections was 2.97 months (IQR 2.29–4.47). Detailed information on each patient’s treatment and follow-up (e.g. treatment type and duration, time of blood collection, recurrence and surgery) is displayed in Fig. 1e, f, and Supplementary Fig. 1.

**Table 1:** Patient-level and blood-level demographic (age and sex) and clinicopathologic (AJCC staging, primary tumour location, treatment type and treatment response) details

Characteristic	Patient-level (all patients n = 29)	Blood-level (during-treatment blood collections n = 56)
Age at diagnosis, median years (IQR)	56 (36–64)	N/A
Age at time of blood collection, median years (IQR)	N/A	55 (35–65)
Sex, n (%)		
Male	13 (44.8)	20 (35.7)
Female	16 (55.2)	36 (64.3)
Stage (AJCC) (%)		
II	5 (17.2)	7 (12.5)
III	15 (51.7)	18 (32.1)
IV	9 (31.0)	31 (55.4)
Location of primary tumour (%)		
Proximal colon	7 (24.1)	21 (37.5)
Distal colon	7 (24.1)	11 (19.6)
Rectosigmoid	3 (10.3)	6 (10.7)
Rectum	12 (41.4)	18 (32.1)
Treatment type (%)		
Chemotherapy only	9 (31.0)	20 (35.7)
Chemotherapy + targeted therapy^1^	11 (37.9)	31 (55.4)
Chemotherapy + radiotherapy^2^	9 (31.0)	1 (1.79)
Targeted therapy only	0 (0)	4 (7.14)
Response status^3^		
Complete responders (%)	12 (41.4)	17 (30.4)
Partial responders (%)	7 (24.1)	20 (35.7)
Stable patients (%)	1 (3.45)	10 (17.9)
Progressors (%)	1 (3.45)	9 (16.1)
Combination (%)	8 (27.6)	N/A

^1^Chemotherapy and targeted therapy either separately or concurrently

^2^Chemotherapy and radiotherapy either separately or concurrently

^3^For patients: overall treatment response observed at all blood collections (e.g. “complete responders” had a complete response at all blood collections, “combination” had varying responses at different blood collections). For blood collections: treatment response observed at the time of any one blood collection

The median age of the patient cohort at diagnosis was 56 years (IQR 36–64 years) and 55.2% of patients were female. 51.7% of patients had stage III CRC at diagnosis, 41.4% of patients were diagnosed with rectal cancer, and 41.4% of patients demonstrated an overall complete response to treatment.

The median age at time of blood collection was 55 years (IQR 35–65 years) and 64.3% of blood collections were from females. 55.4% of blood collections were from patients with stage IV CRC, 37.5% were from patients with proximal colon cancers and 41.4% were from patients with a partial response to treatment at time of collection.

### ctDNA positivity as a predictor of CRC disease presence during treatment

Of the 56 blood samples collected during treatment, 50.0% were ctDNA positive and 80.4% were CEA positive (Table 2). ctDNA positivity at any point in time during treatment was significantly associated with presence of disease (82.9%) compared to the absence of disease (17.1%, *p* = 0.042). Patients with stage IV disease were significantly more likely to have disease present during treatment (87.1%) compared to those with stage II/stage III disease (48.0%, *p* = 0.003). Patients who underwent targeted therapy only, chemotherapy + targeted therapy, or chemotherapy + radiotherapy at time of blood collection were significantly more likely to have disease present during treatment (86.1%) compared to those who underwent chemotherapy only (40.0%, *p* = 0.0006).

**Table 2:** Univariate analysis of factors associated with CRC disease presence during treatment at time of a blood test

Characteristic		Disease present		
		No	Yes	*p*-value
Age^3^	< 55 years (%)	8 (26.7)	22 (73.3)	0.519^2^
	≥ 55 years (%)	9 (34.6)	17 (65.4)	
Sex	Male (%)	6 (30.0)	14 (70.0)	0.965^2^
	Female (%)	11 (30.6)	25 (69.4)	
Stage	Stage II–III (%)	13 (52.0)	12 (48.0)	0.003^1^
	Stage IV (%)	4 (12.9)	27 (87.1)	
Treatment type	Chemotherapy only (%)	12 (60.0)	8 (40.0)	0.0006^1^
	Other^4^ (%)	5 (13.9)	31 (86.1)	
ctDNA result	Negative (%)	12 (42.9)	16 (57.1)	0.042^1^
	Positive (%)	5 (17.9)	23 (82.1)	
CEA result	Negative (%)	1 (9.09)	10 (90.9)	0.144^1^
	Positive (%)	16 (35.6)	29 (65.4)	

*p*-values significant at a < 0.05 level are highlighted in bold

^1^Fisher’s exact test

^2^Chi-squared test

^3^Above and below 55 yrs were selected as classifiers, as this was the median age of the cohort

^4^ “Other” is defined as having undergone targeted therapy only, chemotherapy and targeted therapy concurrently, or chemotherapy and radiotherapy concurrently at time of blood collection

However, when adjusted for stage and treatment type, ctDNA positivity was not a significant predictor of disease presence during treatment (Table 3).

**Table 3:** Multivariable binomial logistic regression analysis of ctDNA positivity as a predictor of disease presence during treatment, with adjustment for stage and treatment type

Predictor	Odds ratio	95%CI	*p*-value
ctDNA result			
Negative	1.0 (ref)		
Positive	1.18	0.169–8.28	0.865
Stage			
II–III	1.0 (ref)		
IV	2.02	0.082–50.1	0.667
Treatment type			
Chemotherapy only	1.0 (ref)		
Other	0.195	0.028–1.36	0.099

### Quantitative ctDNA levels as an indicator of treatment response

Although there was no significant difference in ctDNA test positivity, ctDNA levels were significantly higher in patients with disease present compared to those with no disease present (median 0.036%, IQR 0–1.17% vs. median 0%, IQR 0–0.005%; *p* = 0.016; Fig. 1b). Levels of ctDNA were particularly low in patients who had a complete response to treatment, where 70.6% (12/17) of blood samples had no detectable ctDNA. Of blood collected from patients who had detectable ctDNA despite clinicopathologic scans indicating a complete response to treatment, 40.0% (2/5) were collected prior to a recurrence and 1/5 collections was from a patient who underwent surgery and adjuvant treatment, who then passed from septic shock after completion of treatment (Fig. 1e, f). Additionally, 59.0% (23/39) of patients who either partially responded, did not respond or progressed on treatment had detectable ctDNA. CEA levels were not significantly different between any of the different treatment response groups (Fig. 1c, d).

When normalised to pre-treatment levels, ctDNA levels were significantly higher in patients with disease present compared to those without evidence of disease during treatment (*p* = 0.020) (Supplementary Fig. 2b). However, as with absolute ctDNA levels, there was no significant difference of normalised ctDNA levels between complete responders, partial responders, stable patients and progressors (Supplementary Fig. 2a). There was no significant difference in normalised CEA levels between any treatment response group (Supplementary Fig. 2c and 2d).

### Change in ctDNA levels as an indicator of treatment response

**Fig. 2:**
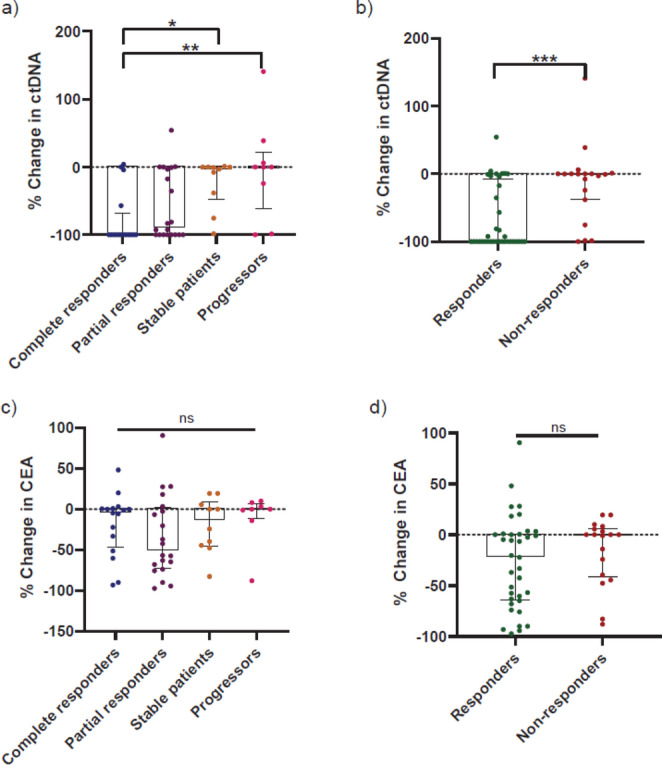
Percentage change in ctDNA and CEA levels between consecutive bloods normalised to pre-treatment measurements. **a** % Change in ctDNA levels, compared between complete responders, partial responders, stable patients and progressors. **b** % Change in ctDNA levels, compared between responders and non-responders. **c** % Change in CEA levels, compared between complete responders, partial responders, stable patients and progressors. **d** % Change in CEA levels, compared between responders and non-responders. Kruskal–Wallis tests were used for comparisons between complete responders, partial responders, stable patients and progressors, with post-hoc Dunn’s test for multiple pairwise comparisons. Mann–Whitney U tests were used for comparisons between responders and non-responders. *p < 0.05, **p < 0.01

The percentage change in ctDNA and CEA levels between consecutive during-treatment blood collections was assessed to determine any differences between treatment response groups, when normalised to pre-treatment measurements (Fig. 2).

The percentage change in ctDNA levels were significantly different between complete responders, partial responders, stable patients and progressors (*p* = 0.003; Fig. 2a), where there was greater reduction in levels in complete responders compared to stable patients and progressors (*p* = 0.022 and *p* = 0.008 respectively). There was a clear trend of greater ctDNA level reductions with improved treatment response. The median and IQR of ctDNA level % change were: − 100% (− 100% to − 67%) in complete responders, − 87.7% (− 100% to − 1.2%) in partial responders, − 1.83% (− 47.4% to 0%) in stable patients and 0% (− 61.4% to 22.5%) in progressors. Additionally, in patients demonstrating any response to treatment (complete or partial), there was a significantly greater reduction in ctDNA levels of − 100% (IQR − 100% to − 7.53%) compared to only − 0.13% (IQR − 38.1% to 0.03%) in patients that did not respond to treatment (stable/progression), *p* = 0.0004 (Fig. 2b). When normalised to pre-treatment measurements, change in CEA levels were unsuccessful in differentiating treatment response groups (*p* > 0.05; Fig. 2c, d).

However, when levels were not normalised to pre-treatment measurements, change in CEA levels were significantly different between different treatment response groups, where progressors had a significant increase in CEA levels compared to complete responders, partial responders and stable patients (*p* = 0.004, *p* < 0.0001 and *p* = 0.0115 respectively) (Supplementary Fig. 3c). The median and IQR of CEA level change were: − 0.10 ng/mL (− 1.5 to 0.15 ng/mL) in complete responders, − 3.60 ng/mL (− 9.80 to 0.50 ng/mL) in partial responders, 0 ng/mL (− 5.70 to 0.95 ng/mL) in stable patients and 17.7 ng/mL (10.3 to 89.0 ng/mL) in progressors, displaying a trend of greater positive change in CEA levels with poorer treatment response. Additionally, there was a significantly greater increase in CEA levels in patients with no response to treatment compared to those with any response to treatment: median and IQR 2.3 ng/mL (− 0.50 to 17.7 ng/mL) and − 1.50 ng/mL (− 6.28 to 0.18 ng/mL) respectively (*p* < 0.01) (Supplementary Fig. 3d).

## Discussion

Despite being the only blood test in clinical practice for assessing CRC treatment response, CEA is a poor biomarker due to low sensitivity and specificity for CRC, representing a gap in clinical care. The methylated *BCAT1/IKZF1* ctDNA blood test has better potential as a CRC treatment response monitoring tool, as it has high sensitivity and specificity for CRC detection and recurrence (Pedersen et al. 2015; Young et al. 2016), and can reflect treatment response when measured before and after treatment for CRC (Symonds et al. 2018). To further assess its usefulness in a CRC treatment response monitoring context, we have examined whether measurements of methylated *BCAT1/IKZF1* ctDNA in blood collected during treatment can better reflect treatment response compared to CEA. Qualitative ctDNA and CEA measures were poor predictors of treatment response—quantitative ctDNA levels and in particular change in levels were the best approach to identifying treatment response. Importantly, quantitative measures of ctDNA could more accurately reflect treatment response relative to CEA. This study supports implementation of the quantitative methylated *BCAT1/IKZF1* ctDNA blood test to monitor treatment response in patients with CRC.

Of the measures tested, reduction in ctDNA levels were the most accurate in identifying responders versus non-responders in patients with CRC undergoing systemic therapy. This is consistent with the work of Gouda et al. (2022), who found in a mixed cancer cohort (which included 121 patients with gastrointestinal tract cancers), that mutation-based ctDNA sampled during systemic treatment could qualitatively distinguish responders from non-responders, with the latter being significantly more likely to have a positive change in ctDNA levels. Our study also found that decreases in ctDNA levels during treatment was greater in complete responders relative to patients with stable disease and those with progressive disease. This is also consistent with the work of Parikh et al. (2020), who found that in a mixed cohort of metastatic gastrointestinal cancer patients (inclusive of 69 CRC patients), there was a significant decrease in tumour-informed, mutation-based plasma ctDNA levels during systemic treatment relative to pre-treatment levels, in partial responders versus stable patients, and in stable patients versus progressors. Overall, the results of this study are in-line with current literature, demonstrating that the ctDNA assay is tumour-agnostic and superior for treatment response monitoring compared to CEA.

Reductions in CEA levels were greater in responders versus non-responders; however, the difference was not as statistically significant as the ctDNA test. Monitoring patients for longer may allow for CEA to better differentiate responders and non-responders, as there is evidence that change in CEA levels 12–18 months into systemic treatment correlates well with CRC treatment response, and CEA levels can surge early into treatment in responders (Yu et al. 2018; Locker et al. 2006). Despite being inferior to the ctDNA test in identifying responders and non-responders, a change in CEA levels may still be a good indicator of disease progression, with highly significant increases in CEA levels observed in progressors compared to other treatment response groups. This is in line with the standard clinical use of CEA, with elevated CEA levels during treatment being an indicator of possible CRC progression (Locker et al. 2006).

This study showed that to more accurately reflect treatment response, normalisation of the change in ctDNA levels between consecutive blood collections during treatment to the pre-treatment measurements was required to more accurately reflect treatment response, but not for CEA. Previous work has shown that plasma methylated *BCAT1/IKZF1* ctDNA levels and positivity are positively correlated with increasing tumour burden and AJCC stage at diagnosis (Symonds et al. 2022). In contrast, evidence for a relationship between plasma CEA and CRC tumour burden is conflicting and conditions comorbid to CRC can affect CEA levels (Hao et al. 2019; Hermunen et al. 2018; Topdagi and Timuroglu 2018; Xie et al. 2024; Gheybi et al. 2022; Fowler et al. 2020). CEA levels likely do not reflect tumour burden as accurately as ctDNA assays, which detect more cancer-specific changes (Litvak et al. 2014; Gheybi et al. 2022; Fowler et al. 2020). As our cohort had stage II-IV CRC (i.e. differing tumour burden at diagnosis), this may account for why normalisation of ctDNA level changes to pre-treatment measurements is required to differentiate treatment response groups, but not for CEA.

In addition to quantitative change in ctDNA levels, the absolute levels measured during treatment could also identify treatment response. Patients with disease present during treatment had significantly higher ctDNA levels than those without, and ctDNA levels were a good indicator of a lack of disease, with only 5/17 (29%) of blood collections from patients without disease having detectable ctDNA levels. There were plausible reasons for this in 3 cases: 2 collections were prior to CRC recurrence, despite clinicopathologic scans at the time of blood collection indicating no disease, and 1 case was from a patient who passed from septic shock after treatment completion. Patients with sepsis/septic shock have differential methylation in genes linked with immune cell differentiation and activation (such as *BCAT1* and *IKZF1*) (Beltrán-García et al. 2024; Boutboul et al. 2018; Ananieva et al. 2014), and increased levels of cfDNA (Charoensappakit et al. 2023). Therefore, the elevated methylated *BCAT1/IKZF1* cfDNA levels could be due to the onset of sepsis rather than the presence of CRC. In contrast to the ctDNA assay, quantitative CEA levels were unsuccessful in differentiating any treatment response group. This is unsurprising, as *elevation* in post-operative CEA levels may indicate cancer progression, rather than the absolute values itself (Locker et al. 2006).

While the quantitative ctDNA levels and change in levels during treatment were useful in identifying treatment response, qualitative measures were not. Though univariate analysis suggested an association between disease presence and ctDNA test positivity during treatment, this was lost when adjusted for the presence of metastatic disease. This indicates that ctDNA test positivity was mainly driven by having metastatic disease, and indeed the frequency of our assay’s positivity increases with stage (Pedersen et al. 2015). CEA positivity during treatment was also unsuccessful in identifying the presence of CRC, which may be due to its low specificity (Hao et al. 2019).

The study was not without limitations. To minimise patient burden, blood collection and radiological imaging was conducted opportunistically at medical appointments scheduled as part of clinical care. However, ctDNA release is highly dynamic, and can vary with the time of day (Tóth et al. 2017). Therefore, opportunistic blood collection could affect the accuracy of the test. The small sample size combined with diversity of disease stage was another limitation. Considering that the association of ctDNA test positivity during treatment with disease presence was lost when adjusted for the presence of metastatic disease, the assay may perform better in a metastatic CRC cohort. This is unsurprising, since is known that positivity of the assay is associated with advancing CRC stage (Symonds et al. 2022). However, the small sample size did not allow for subgroup analysis. Larger studies are warranted to allow for investigation of assay performance in different stages of CRC. Additionally, only patients who were ctDNA-positive pre-treatment were included, for more precise assessment of the assay’s ability to reflect treatment response (i.e. lack of ctDNA treatment can be attributed to treatment response, rather than the lack of baseline detectable ctDNA pre-treatment). However, to be generalisable for all patients undergoing treatment for CRC, it is important to conduct further investigation regardless of ctDNA assay results pre-treatment.

## Conclusion

Overall, this study has demonstrated superior efficacy of the methylated *BCAT1/IKZF1* ctDNA test to predict real-time treatment response in stage II-IV CRC patients undergoing cancer treatment compared to CEA, which is the current blood biomarker test used in clinical care. Quantitative measures of our ctDNA assay proved most useful in identifying treatment response in our patient cohort, whereas a binary ctDNA test was less effective. The results also indicate better suitability of our assay for treatment response monitoring in a metastatic CRC cohort; therefore, further observational research with a larger sample size and stratification into disease stage is required. Possible future integration of the ctDNA test into CRC treatment monitoring protocols could involve use as a supplementary non-invasive test to radiological imaging. ctDNA levels can inform response or non-response to treatment, which could potentially be used to facilitate an early switch to alternative therapies in non-responsive cases, provide additional assurance of treatment efficacy in responsive cases, or streamline radiological imaging intervals to reduce radiation exposure while still ensuring effective treatment response monitoring. However, application in a clinical setting would require additional validation of observational data with randomised controlled trials.

Overall, using the methylated *BCAT1/IKZF1* ctDNA blood test could improve patient outcomes by personalising treatment strategies in a timely manner to ensure the most robust treatment outcomes, and consequently reduce recurrence and mortality due to CRC.

## Supplementary Information

Below is the link to the electronic supplementary material.

Supplementary Material 1

## Data Availability

The datasets analysed in this study are available from the corresponding author upon request.
