# Supplementary material for: Investigation of a methylated BCAT1 and IKZF1 circulating tumour DNA blood test for monitoring treatment response in patients with colorectal cancer
Source: J Cancer Res Clin Oncol. 2026 Jun 2;152(8):156. doi: 10.1007/s00432-026-06512-x (PMC13442765; doi:10.1007/s00432-026-06512-x)
Supplement: Supplementary file 1 [file 432_2026_6512_MOESM1_ESM.docx]

**Supplementary Information**

**Supplementary Figure 1.** Individual patient methylated *BCAT1* ctDNA levels, methylated *IKZF1* ctDNA levels, average methylated *BCAT1/IKZF1* ctDNA levels and CEA levels across each patient’s treatment. Methylated *BCAT1=*dark blue circle, methylated *IKZF1=*yellow circle, average methylated *BCAT1/IKZF1*=green circle, black triangle=CEA. Periods of chemotherapy ± targeted therapy and targeted therapy only are shaded in light grey and periods of radiotherapy are shaded in dark grey. Surgery is represented by a green dotted line, recurrence is represented by a red dotted line and death is represented by a black dotted line. Treatment response status is indicated above each ctDNA blood collection point (CR=complete response, PR=partial response, SD=stable disease and PD=progressive disease.

**Supplementary Figure 2**. a) ctDNA levels during treatment in complete responders, partial responders, stable patients and progressors (normalised to pre-treatment levels, expressed as a percentage). b) ctDNA levels during treatment in patients with disease present vs. no disease (normalised to pre-treatment levels). c) CEA levels during treatment in complete responders, partial responders, stable patients and progressors (normalised to pre-treatment levels). d) CEA levels during treatment in patients with disease present vs. no disease (normalised to pre-treatment levels).

**Supplementary Figure 3**. Change in ctDNA and CEA levels between consecutive bloods. a) Change in ctDNA levels, compared between complete responders, partial responders, stable patients and progressors (% pg/*ACTB*). b) % Change in ctDNA levels, compared between responders and non-responders (% pg/*ACTB*). c) % Change in CEA levels, compared between complete responders, partial responders, stable patients and progressors (ng/mL). d) % Change in CEA levels, compared between responders and non-responders (ng/mL). Kruskal-Wallis tests were used for comparisons between complete responders, partial responders, stable patients and progressors, with post-hoc Dunn’s test for multiple pairwise comparisons. Mann-Whitney U tests were used for comparisons between responders and non-responders. **p*<0.05, ***p*<0.01, *****p*<0.0001
